# Integrating in vitro data and physiologically based kinetic (PBK) modelling to assess the in vivo potential developmental toxicity of a series of phenols

**DOI:** 10.1007/s00204-016-1881-x

**Published:** 2016-11-04

**Authors:** Marije Strikwold, Bert Spenkelink, Laura H. J. de Haan, Ruud A. Woutersen, Ans Punt, Ivonne M. C. M. Rietjens

**Affiliations:** 1 Division of Toxicology, Wageningen University, Stippeneng 4, 6708 WE Wageningen, The Netherlands; 2 Van Hall Larenstein University of Applied Sciences, PO Box 1528, 8901 BV Leeuwarden, The Netherlands; 3 TNO Innovation for Life, PO Box 360, 3700 AJ Zeist, The Netherlands; 4 WUR/TNO Centre for Innovative Toxicology, PO Box 8000, 6700 EA Wageningen, The Netherlands

**Keywords:** Physiologically based kinetic (PBK) modelling, In vitro–in vivo extrapolation (IVIVE), Alternative for animal testing, Substituted phenols, Reverse dosimetry, Embryonic stem cell test (EST)

## Abstract

Toxicity outcomes derived in vitro do not always reflect in vivo toxicity values, which was previously observed for a series of phenols tested in the embryonic stem cell test (EST). Translation of in vitro data to the in vivo situation is therefore an important, but still limiting step for the use of in vitro toxicity outcomes in the safety assessment of chemicals. The aim of the present study was to translate in vitro embryotoxicity data for a series of phenols to in vivo developmental toxic potency values for the rat by physiologically based kinetic (PBK) modelling-based reverse dosimetry. To this purpose, PBK models were developed for each of the phenols. The models were parameterised with in vitro-derived values defining metabolism and transport of the compounds across the intestinal and placental barrier and with in silico predictions and data from the literature. Using PBK-based reverse dosimetry, in vitro concentration–response curves from the EST were translated into in vivo dose–response curves from which points of departure (PoDs) were derived. The predicted PoDs differed less than 3.6-fold from PoDs derived from in vivo toxicity data for the phenols available in the literature. Moreover, the in vitro PBK-based reverse dosimetry approach could overcome the large disparity that was observed previously between the in vitro and the in vivo relative potency of the series of phenols. In conclusion, this study shows another proof-of-principle that the in vitro PBK approach is a promising strategy for non-animal-based safety assessment of chemicals.

**Electronic supplementary material:**

The online version of this article (doi:10.1007/s00204-016-1881-x) contains supplementary material, which is available to authorized users.

## Introduction

The safety evaluation of chemicals is currently evolving from using animal toxicity tests towards the application of innovative non-animal based in vitro approaches to predict toxicity. This development is encouraged by initiatives such as the European Registration, Evaluation, Authorisation and restriction of CHemicals (REACH) (EC 2007), the Cosmetic Products Regulation (EC 2009) and the US National Research Council report on toxicity testing in the twenty-first century (National Research Council 2007). For many years, in vitro toxicity assays have been used for hazard identification only, including the detection of genotoxicity and the ranking and prioritisation of compounds for further in vivo toxicity testing (Gülden and Seibert 2005). Translation of in vitro data to the in vivo situation is an important but limiting step for the use of in vitro outcomes in the regulatory risk assessment of chemicals, as toxicity outcomes in vitro derived as such do not always reflect in vivo toxicity values (Blaauboer 2010; Punt et al. 2011). For example, in our study on the embryotoxic potencies of a series of phenols evaluated in vitro with the ES-D3 differentiation assay of the embryonic stem cell test (EST), it was concluded that the assay did not correctly rank the phenols according to their in vivo potency (Strikwold et al. 2012). In particular, the toxicity of *p*-heptyloxyphenol was relatively higher in the EST than reported in in vivo studies in the literature as compared to the other phenols tested. In the EST, *p*-heptyloxyphenol displayed a BMC_50_ that was more than three orders of magnitude lower than that of phenol whereas in vivo BMD_10_ values differed less than threefold (Strikwold et al. 2012). Kinetic differences between the in vitro and in vivo situation were hypothesised to provide a reason for the observed disparities (Strikwold et al. 2012).

Combining in vitro toxicity data with physiologically based kinetic (PBK) modelling applying reverse dosimetry has recently been shown to provide a promising approach to extrapolate in vitro concentration–response curves to in vivo dose–response curves from which points of departure (PoDs) for the risk assessment of chemicals can be derived (Louisse et al. 2010, 2015; Strikwold et al. 2013). In this way kinetic differences between the vitro and in vivo situation can be taken into account and in vivo dose–response curves suitable for deriving a PoD for risk assessment can be obtained based on in vitro data. However, as PBK models are generally data intense and their development is often time consuming (Loizou and Hogg 2011), their application is often hampered. Applying in silico predictions, i.e. quantitative structure–activity relationships (QSARs) and in vitro kinetic experiments may aid the development of PBK models by predicting input values for kinetic parameters required, thereby also facilitating non-animal-based safety assessment of chemicals.

For phenol, one of the congeners tested in our previous in vitro study on the embryotoxic potency of a series of phenols (Strikwold et al. 2012), in vitro PBK-based reverse dosimetry and subsequently dose–response modelling, provided a PoD that appeared to match the range of in vivo-derived PoDs (Strikwold et al. 2013). The aim of the present study was to translate in vitro embryotoxicity data for a series of phenols including *p*-heptyloxyphenol as previously obtained with the EST (Strikwold et al. 2012), to in vivo developmental toxicity values for the rat by PBK-based reverse dosimetry, using in silico- and in vitro-defined kinetic parameters. Ultimately, this should elucidate whether combining in vitro data with PBK modelling to predict in vivo values can overcome differences that were observed between the in vitro and the in vivo relative potencies of different phenolic congeners, and especially whether this approach can overcome the deviating results for *p*-heptyloxyphenol. This may provide another proof of principle to assess the feasibility of this in vitro PBK approach for prospective toxicological safety evaluations of chemicals.

## Materials and methods

### Compounds and materials

Phenol (99%), *p*-fluorophenol (99%), *p*-heptyloxyphenol (97%), *p*-methylketophenol (99%), antipyrine (≥99%), fluorescein, Tris(hydroxymethyl)aminomethane (Tris) (≥99.9%) and Dulbecco’s modified Eagle’s medium (DMEM), uridine 5′-diphosphoglucuronic acid (UDPGA), alamethicin (98%), sodium taurocholate hydrate (97%), *β*-glucuronidase (Type 1x-A from *Escherichia coli*) and bovine serum albumin (≥98%) were obtained from Sigma-Aldrich (Steinheim, Germany). Acetonitrile (ULC/MS grade) and methanol (HPLC supra-gradient) were obtained from BioSolve (Valkenswaard, the Netherlands), and dimethylsulfoxide (DMSO) (≥99%) from Acros Organics (Geel, Belgium). Trifluoroacetic acid (TFA), hydrochloric acid (37%), magnesium chloride hexahydrate, potassium phosphate (≥99%), and Transwell^®^ inserts (0.4 µm pored polycarbonate membrane, 12 mm diameter) were purchased from VWR International GmbH (Darmstadt, Germany). Foetal calf serum (FCS) was purchased from HyClone-Perbio (Etten-Leur, the Netherlands). Penicillin, streptomycin, l-glutamine, minimal essential medium non-essential amino acids and trypsin/EDTA in PBS (final concentration 0.025/0.01%) were obtained from Gibco (Paisley, Scotland). Phosphate-buffered saline (PBS) and Hank’s balanced salt solution (HBSS) were obtained from Invitrogen (Breda, the Netherlands) and HEPES was from VWR (Radnor, USA).

BeWo choriocarcinoma cells subclone b30 were kindly provided by the Institute of Public Health of the Faculty of Health Sciences of the University of Copenhagen (Denmark) with permission from Dr. Alan Schwartz (Washington University, St Louis, MO, USA). The provided cell line was confirmed to be mycoplasma negative. The colorectal adenocarcinoma (Caco-2) cells were obtained from ATCC (Middlesex, UK). Pooled liver microsomes from male Sprague–Dawley rats were obtained from BD Biosciences Gentest (Woburn, MA, USA).

### General outline in vitro PBK approach

The in vitro PBK approach to predict in vivo dose–response curves and a PoD for risk assessment using in vitro embryotoxicity data consisted of the following steps: (1) establishment of in vitro effective concentrations (EC_*x*_) of the phenols in the EST, (2) development of PBK models describing in vivo kinetic properties of the phenols in rat including derivation of PBK model parameters, (3) sensitivity analysis of the PBK models, (4) translation of in vitro EC_*x*_ values into in vivo external dose levels (ED_*x*_) generating dose–response curves for developmental toxicity in rat and enabling the definition of a PoD for risk assessment and (5) evaluation of predictions performed with the in vitro PBK approach.

### In vitro embryotoxicity

Embryotoxicity data of phenol, *p*-fluorophenol, *p*-heptyloxyphenol and *p*-methylketophenol determined using the murine embryonic stem cell (ES-D3) differentiation assay of the EST by Strikwold et al. (2012) were used as a starting point for the in vitro PBK approach translating in vitro embryotoxicity data to in vivo toxicity values. The use of both the maximum concentration in foetal plasma (*C*_max_) and the area under the foetal plasma concentration–time curve (AUC) in the in vitro PBK approach was evaluated. The concentration of phenol and *p*-fluorophenol decreased in time in the EST culture medium (without cells) in a similar fashion, while *p*-methylketophenol and *p*-heptyloxyphenol were stable (Strikwold et al. 2012). Therefore, the AUC of the 10-day EST of *p*-methylketophenol and *p*-heptyloxyphenol was calculated by multiplying the test concentration by the duration of the experiment (AUC_0–10d_), while the calculated AUC_0–10d_ for phenol and *p*-fluorophenol was reduced by 39% corresponding to the loss of phenol in the EST as reported by Strikwold et al. (2013).

### Rat PBK model structures for phenols

**Fig. 1:**
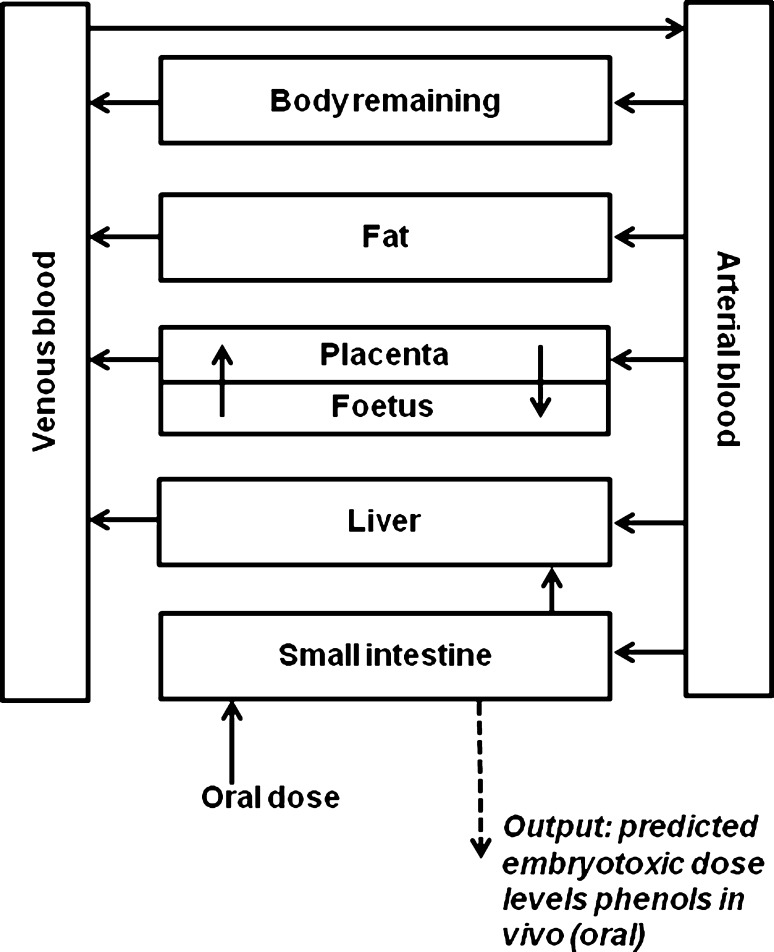
Schematic representation of the PBK models of the phenols

The rat PBK model for phenol developed by Strikwold et al. (2013) was used as a starting point to construct rat PBK models for the different phenols of the present study (Fig. 1), with four major modifications. The first modification is that only liver glucuronidation of the phenolic compounds was taken into account in the present study to describe the metabolic conversions. This could be done because the sensitivity analysis of the previously developed PBK model of phenol by Strikwold et al. (2013), performed at an oral dose level of 150 mg/kg bw which is consistent with high oral dose levels that were applied in in vivo toxicity studies of the phenols (Kavlock 1990), identified that glucuronidation of phenol in the liver is the most influential metabolic pathway in the model. Moreover, in vivo kinetic studies towards the metabolism of phenol confirm the importance of this route, showing that the glucuronide conjugate is the predominant metabolite formed at high oral dose levels (Hiser et al. 1994). Furthermore, the metabolic parameters *K*_m_ and *V*_max_ of sulfation were not identified as a sensitive parameter in the PBK model for phenol at high oral dose levels, and the *V*_max_ value for sulfation was very low (Strikwold et al. 2013), supporting the choice for glucuronidation as the metabolic and elimination pathway in the current PBK models. A second modification is that we have included in vitro transport experiments with Caco-2 cells to define the oral uptake constants of the different compounds of the present study, since the previously predicted plasma concentrations of phenol appeared to be quite sensitive to the oral absorption coefficient (ka). A third modification is that a fat compartment was included in the PBK models for the different phenolic compounds, because *p*-heptyloxyphenol may readily be distributed to adipose tissue due to its relatively high lipophilicity. Finally, a placental/foetal compartment was added to the PBK models, including transport of the compound from the mother to the embryos/foetuses and back by simple diffusion. For the placental/foetal compartment, the number of embryos in one litter was considered to be 12, and they were treated as one unit for which the physiological parameters were calculated. Foetal–maternal diffusion was set equal to maternal–foetal diffusion. To include maternal–foetal diffusion in the PBK model, apparent permeability coefficients (*P*_app_) across placental BeWo cells cultivated in a transwell system were derived in vitro and subsequently converted to in vivo diffusion transplacental clearance rates (l/h; see “In vitro placental transport study” section). The PBK models were defined with parameters representative for gestational day 11 (GD11), facilitating evaluation of the PBK model predictions with available in vivo developmental toxicity data for rats exposed to phenol or *p*-substituted phenol at GD11.

An overview of the PBK model algorithms, which are similar for all the phenols, is included in Supplementary data A. Kinetic model calculations were performed by applying Rosenbrock’s algorithms for solving stiff systems (Berkeley Madonna, version 8.3.18, UC Berkeley, CA, USA).

### PBK model parameter values

#### Physiological parameters

The physiological parameters used in the PBK model of phenol in the rat of Strikwold et al. (2013) were used as starting point to define parameters for the pregnant rat at GD11. In the PBK models for the phenols, the volume of the liver, adipose tissue, uterus, mammary gland, placentas and foetuses were estimated by the gestational growth algorithms of O’Flaherty et al. (1992). The uterus and mammary gland were not included as specific compartments in the model and the change in volume of these compartments was taken into account in the remaining body compartment. The volume of the foetuses included also the amniotic fluid volume of GD11 that was reported by Park and Shepard (1994) and Fisher et al. (1993), because at this gestational day maternal–foetal exchange occurs via both the chorioallantoic placenta and the yolk sac placenta (Carney et al. 2004). The volume of the intestine, venous and arterial blood was kept similar to values for a non-pregnant rat. The total maternal body weight was the sum of the body weight of a non-pregnant rat and the placentas and foetuses as well as the change of the volume of the liver, adipose tissue, uterus and mammary gland during pregnancy.

The total cardiac output and the blood flow to the liver for GD11 were obtained from Buelke-Sam et al. (1982a, b). The blood flow to the placentas was estimated by the gestational growth algorithms of O’Flaherty et al. (1992). The blood flow to the adipose tissue was proportional to the relative weight of the adipose tissue. The blood flow to the small intestine was calculated using reported blood flow rates for the specific parts of the splanchnic system of the non-pregnant rat by Delp et al. (1998). All physiological parameters are presented in Table 1.

**Table 1:** Physiological data for the rat applied in the PBK models of the phenols

Parameter		
Model compartments	Percentage of body weight^a^	Percentage of cardiac output
Small intestine	1.3	5.7
Liver	3.6	17.3^b^
Adipose tissue	7.8	6.3
Arterial blood	1.8	
Venous blood	5.3	
Placentas	0.30	0.26
Foetuses and amniotic fluid	0.13	
Body remaining	71.2	70.5
	l/h	
Cardiac output	7.1	

^a^Organ percentages of total maternal body weight at GD11

^b^Without flow from small intestine

#### In silico predictions of physicochemical and biochemical parameters

An overview of physicochemical parameters that are used to predict biochemical and distribution parameters of the compounds are included in Table 2. Tissue plasma partition coefficients (Pt:p) were calculated using the algorithm of Berezhkovskiy (2004), which requires information on plasma protein binding, lipophilicity and acid–base properties (see Tables 2, 3). The adipose tissue–plasma partition coefficients were calculated using the olive oil–water distribution coefficient (*D* × vo:w_pH7.4_), and the partition coefficients for the non-adipose tissues were calculated with the n-octanol–water partition coefficient (Pow) for the non-ionised species at pH 7.4. The value of *D* × vo:w_pH7.4_ was calculated using log Po:w and pKa according to the algorithms reported by Poulin and Theil (2002). The unbound fraction of the compound in plasma was calculated with the Simcyp model (Simcyp 2015), after which these values were converted to the unbound fraction in tissue using the algorithm of Poulin and Theil (2002). The Pt:p values of the placental compartment were set equal to the predicted liver–plasma partition coefficient and the Pt:p of the embryonic/foetal unit was a volume-weighted whole body–plasma partition coefficient, in which the adipose tissue was omitted because of the low amount of fat in foetal rat (Sarr et al. 2012). Partitioning of the compounds between red blood cells and plasma (Prbc:p) was predicted with the algorithms of Paixão et al. (2009) and was subsequently used to calculate the partition coefficient of the compounds between blood and plasma (Pb:p) using the following equation:

$${\text{Pb:p}} = ({\text{Prbc:p}} \times {\text{HTC}}) + (1 - {\text{HTC}})$$

where HTC corresponds to a hematocrit fraction of 0.45.

**Table 2:** Physicochemical properties of the phenols

Parameter	Phenol	*p*-Fluoro	*p*-Heptyloxy	*p*-Methylketo
MW (Da)^a^	94.11	112.10	208.30	136.15
Log Po:w^a^	1.54	1.84	4.41	1.40
pKa^a^	9.86	9.92	10.35	8.12
% Ionised in serum (pH 7.4)^b^	0	0	0	16

^a^Values from ACD/Labs (2015)

^b^Calculated with Simcyp model (Simcyp 2015)

**Table 3:** In silico-predicted biochemical and distribution parameters of the phenols for the rat

Parameter	Phenol	*p*-Fluoro	*p*-Heptyloxy	*p*-Methylketo
Fu (pH 7.4; –)^a^	0.38	0.31	0.03	0.28
Partition coefficients^b^				
Liver–plasma	0.81	0.96	7.99	0.64
Intestine–plasma	0.97	1.20	11.57	0.74
Adipose–plasma	0.83	1.42	84.38	0.39
Placenta–plasma^c^	0.81	0.96	7.99	0.64
Foetus–foetal plasma^d^	0.76	0.89	6.92	0.61
Body remaining–plasma	0.75	0.87	6.67	0.61
Blood–plasma^e^	0.72	0.70	1.48	0.67

^a^Fraction unbound to plasma proteins (Fu). Calculated with Simcyp model (Simcyp 2015)

^b^Calculated with algorithms of Berezhkovskiy (2004)

^c^Assumed to be the same as the partition coefficient of the liver

^d^Whole body–plasma partition coefficient without adipose tissue (volume weighted)

^e^Calculated with algorithms of Paixão et al. (2009)

#### In vitro intestinal transport study

Caco-2 cells (passages 37–40) were cultured in DMEM containing 25 mM HEPES (pH 7.4) supplemented with 10% (v/v) heat-inactivated FCS, 4500 mg/l glucose, 2 mM l-glutamine, 1% (v/v) minimal essential medium non-essential amino acids, 10,000 U/ml penicillin and 10 mg/ml streptomycin and maintained in polystyrene cell culture flasks (Corning, Amsterdam, the Netherlands) in a 5% CO_2_-humidified atmosphere at 37 °C. Cells were harvested after exposure to a trypsin–EDTA solution. Next, the cells were seeded onto Transwell^®^ inserts (0.4 µm pored polycarbonate membrane, 12 mm diameter), at a density of 10^5^ cells/cm^2^. Cell culture medium (0.5 and 1.5 ml in the apical and basolateral compartment, respectively) was changed every two days.

Compounds that were included in the transport experiments were phenol, *p*-fluorophenol, *p*-heptyloxyphenol, *p*-methylketophenol, antipyrine (passive transcellular control) and fluorescein (passive paracellular control) as well as DMSO (solvent control). Stock solutions of the test compounds were prepared in DMSO and tested at a final DMSO level in the transport buffer of 0.2%, except for *p*-heptyloxyphenol for which the final DMSO level was 0.5% due to the relatively lower solubility of this compound.

Transport experiments were performed between day 20 and 23 post-seeding. Prior to the transport experiments, the cell culture medium was removed and cells were equilibrated in HBSS for 30–45 min in a 5% CO_2_-humidified atmosphere at 37 °C. In these 45 min, the integrity of the cell monolayer was examined by measuring the Trans Epithelial Electrical Resistance (TEER) of the cell layer using a Millicell ERS-2 Volt–Ohm Meter (Millipore, USA). Only cell layers with a TEER value between 500 and 1000 Ω cm^2^ were used for the transport experiments. The transport buffer for the apical compartment consisted of HBSS containing 10 mM HEPES (pH 6.5) and 10 mM sodium taurocholate and the transport buffer for the basolateral compartment consisted of HBSS (pH 7.4) with 30 mg/ml bovine serum albumin. At first, 1.5 ml pre-warmed (37 °C) transport buffer was added to the basolateral compartment. Then, the transport experiments were started by adding 0.5 ml pre-warmed transport buffer containing the test compound (100 µM) to the apical compartment. After 60-min incubation in a 5% CO_2_-humidified atmosphere at 37 °C, a 75 µl sample was taken from the basolateral compartment and then from the apical compartment. Each sample was added to 150 µl ice-cold methanol, vortexed and put on ice immediately. The filters of the Transwell^®^ inserts were washed one time with HBSS and two times with PBS, then cut out from the insert and added to 250 µl methanol 65% (v/v) and sonificated for 15 min by a Bandelin Sonorex RK100 sonificator (Berlin, Germany). Samples were analysed by UPLC–PDA (see “Quantification of analytes” section) for the presence of the test compound and possible metabolites.

TEER values were also measured at the end of the transport experiment, during the first washing step and compared to the TEER values measured before the transport experiment in order to assess toxicity of the test compound to the cell monolayer. Caco-2 monolayers were omitted from further analysis when the TEER value was reduced more than 15% during the transport experiments (Wang et al. 2014). The Caco-2 transport studies encompassed four independently performed experiments including three replicates in each assay.

The apparent permeability coefficients (*P*_app_; cm/s) were calculated using the following algorithm:

$$P_{\text{app}} = \frac{{\frac{\Delta Q}{\Delta t}}}{{A * C_{0} }}$$

where *P*_app_ is the apparent permeability coefficient (cm/s), Δ*Q*/Δ*t* (nmol/s) is the amount of the test compound transported to the receiver chamber in a certain time period, *A* is the transwell membrane surface area (cm^2^) and *C*_0_ is the initial concentration of the test compound in the donor compartment (µM).

In order to assess the validity of the *P*_app_ calculations for this experiment, the linearity of transport from the apical to the basolateral side was verified by taking samples from the basolateral side at *t* = 15, 30, 60 and 90 min, at a test concentration of 100 µM. The amount of sample (50 µl) withdrawn from the basolateral side was replaced by a similar amount of Caco-2 transport medium, which was accounted for in assessing the linearity. The recovery of the test compound in each transport experiment was calculated with a mass balance equation, taking into account the amount of the test compound in the apical and basolateral compartment and the amount in the cells and/or filter of the Transwell^®^ insert.

To extrapolate the in vitro-derived apparent permeability coefficients (*P*_app_) from the Caco-2 transport experiments to in vivo oral absorption coefficients (ka), relative *P*_app_ ratios were calculated with phenol as the standard compound (*P*_app_ *p*-substituted phenol/*P*_app_ phenol), which were subsequently multiplied by the ka value for phenol of 7.62/h obtained from the rat in situ intestinal perfusion study of Humphrey et al. (1980).

#### In vitro placental transport study

BeWo cells (passages 28–31) were cultured in DMEM with 4500 mg/ml glucose and supplemented with 10% (v/v) heat-inactivated FCS, 2 mM l-glutamine, 10,000 U/ml penicillin and 10 mg/ml streptomycin and maintained in polystyrene cell culture flasks (Corning, Amsterdam, the Netherlands) in a 5% CO_2_-humidified atmosphere at 37 °C. Cells were harvested after exposure to a trypsin–EDTA solution. Next, the cells were seeded onto Transwell^®^ inserts (0.4-µm pored polycarbonate membrane, 12 mm diameter), with a density of 10^5^ cells/cm^2^. Cell culture medium (0.5 and 1.5 ml in the apical and basolateral compartment, respectively) was changed daily.

Transport experiments were performed 6 days post-seeding. The transport buffer consisted of HBSS with 30 mg/ml bovine serum albumin (apical compartment) and 10 mg/ml bovine serum albumin (basolateral compartment) representing mid- and late gestational maternal and embryonic/foetal rat plasma albumin levels, respectively (Honda et al. 2008; Mcmullin et al. 2008; Yeoh and Morgan 1974). The transport experiments were performed for the same compounds and according to the same method as described for the Caco-2 transport experiments, except that only cell layers with a TEER value >190 Ω cm^2^ were used for the transport experiments. The BeWo transport studies encompassed four independently performed experiments including two replicates in each assay. Samples were analysed by UPLC–PDA (see “Quantification of analytes” section) for the presence of the test compound and possible metabolites. The apparent permeability coefficients were calculated using the same method as described for the Caco-2 transport experiments.

To extrapolate the in vitro-derived apparent permeability coefficients (*P*_app_) obtained from the BeWo transport experiments to in vivo transplacental clearance rates (CLPL), relative *P*_app_ ratios were calculated with antipyrine as the standard compound [*P*_app_ (*p*-substituted) phenol/*P*_app_ antipyrine], which were subsequently multiplied by the transplacental maternal–foetal antipyrine clearance rate of 0.18 l/h. This antipyrine clearance rate was obtained by converting the reported antipyrine transplacental clearance value of 0.448 l/h/kg for the rat at GD20 (Varma and Ramakrishnan (1985) to a value for GD11 via allometric scaling to maternal body weight (O’Flaherty et al. 1992) using a maternal body weight for GD11 reported by Buelke-Sam et al. (1982b).

#### In vitro assays for glucuronidation of phenols by rat tissue

The formation of glucuronide metabolites of the *p*-substituted phenols was investigated in incubations with rat liver microsomes. Incubation mixtures consisted of 50 mM Tris–HCl (pH 7.4) and 10 mM MgCl_2_, containing (final concentrations) rat liver microsomes (0.2 mg protein/ml) and 2 mM UDPGA. To obtain maximum glucuronidation activity, the microsomes were activated by preincubating the incubation mixture with 0.025 mg/ml alamethicin added from a 200 times concentrated stock solution in methanol, during 15 min on ice (Fisher et al. 2000). Subsequently, the incubations were started after a 1-min preincubation at 37 °C by addition of the substrate from a 200 times concentrated stock solution in DMSO and incubated in a shaking water bath at 37 °C for 45 min. The reactions were terminated by addition of ice-cold acetonitrile (20% v/v). In the blank incubation mixtures, UDPGA was omitted. Samples were analysed by UPLC–PDA (see “Quantification of analytes” section). No reference standards of the glucuronide metabolites were commercially available. Hence, the metabolites were identified as follows. At first, retention times from the UPLC–PDA chromatograms of the parent compound of the blank incubation were compared with the retention times of newly appearing peaks in chromatograms of the incubation mixtures, as the glucuronide conjugates are expected to elute earlier compared with their parent compounds due do their increased hydrophilicity. Secondly, the formation of the suggested glucuronide conjugate was confirmed by enzymatic deglucuronidation with *β*-glucuronidase. For this purpose, a volume of 10 µl of the incubation mixture of the glucuronidation assay (that was not terminated by the addition of ice-cold acetonitrile) was added to 90 µl of 200 mM potassium phosphate (pH 6.2). Then, 4 µl of glucuronidase (200 units/ml) was added, and the mixtures were incubated for 60 min in a shaking water bath at 37 °C. The reactions were terminated by addition of ice-cold acetonitrile (20% v/v) and were put on ice. Control samples were treated under the same conditions, but without glucuronidase.

After identifying the formation of the glucuronide conjugates for the *p*-substituted phenols, incubations with rat liver microsomes were performed to quantify kinetic parameters, which are the maximum enzyme reaction rate (*V*_max_) and the Michaelis–Menten constant (*K*_m_). The conditions of the incubation assays were optimised to obtain linear reaction rates with respect to incubation time and protein concentration and non-limiting cofactor levels were applied. The optimised incubation mixtures consisted of 50 mM Tris–HCl (pH 7.4) with 10 mM MgCl_2_, containing (final concentrations) 10 mM UDPGA and 0.1, 0.01 and 0.05 mg microsomal protein/ml for *p*-fluorophenol, *p*-heptyloxyphenol, and *p*-methylketophenol, respectively. To obtain maximum glucuronidation activity, the microsomes were activated by preincubating the incubation mixture with 0.025 mg/ml alamethicin added from a 200 times concentrated stock solution in methanol, during 15 min on ice (Fisher et al. 2000). Subsequently, the incubations were started after a 1-min preincubation at 37 °C by addition of the substrate from a 200 times concentrated stock solution in DMSO and incubated in a shaking water bath of 37 °C for 10 min for *p*-fluorophenol and 2.5 min for *p*-heptyloxyphenol and *p*-methylketophenol, respectively. The incubation experiments encompassed three or four independently performed experiments for each phenol.

Kinetic constants for the glucuronidation of the *p*-substituted phenols were derived by fitting the data to the standard Michaelis–Menten equation;

$$v = \frac{{V_{max} *\left[ S \right]}}{{K_{m} + [S]}}$$

in which [*S*] represents the substrate concentration, *V*_max_ the maximum velocity and *K*_m_ the Michaelis–Menten constant for the formation of the glucuronide metabolites. Data analysis was accomplished using GraphPad Prism 5.0 software (GraphPad, San Diego, CA, USA). For the PBK model, the in vitro-derived *V*_max_ values from rat liver microsomes were scaled to the in vivo situation using a reported microsomal protein yield of 38 mg/g rat liver (Chiu and Ginsberg 2011). The in vivo *K*_m_ value was assumed to be the same to the in vitro *K*_m_ value. Michaelis–Menten constants for phenol were taken from our previous study (Strikwold et al. 2013). The present study assumed unrestricted metabolism despite the high plasma protein binding of *p*-heptyloxyphenol, because the rapid metabolic turnover is assumed to clear the chemical so avidly that protein binding may not be rate limiting.

### Quantification of analytes

Samples from the BeWo and Caco-2 transport experiments were centrifuged at 13,000 rpm at 5 °C for 15 min. Next 7.5 µl of the supernatant of each sample was analysed by UPLC (Waters Acquity). Samples from the glucuronidation assays were centrifuged at 15,000 rpm at 5 °C for 5 min. Subsequently, 3.5 µl of the supernatant of each sample was analysed by UPLC, except for the incubations with *p*-heptyloxyphenol of which 10 µl was analysed by UPLC. All samples, except those from transport studies with fluorescein, were analysed on a Waters BEH C18 1.7 µm column, 2.1 × 50 mm, with nanopure water (0.1% TFA) (A) and pure acetonitrile (B) applying a gradient elution. The start condition was 100:0 (A:B), changing to 90:10 from 1 to 2 min, then to 10:90 from 2 to 4 min (or from 2 to 3 min when analysing samples for *p*-heptyloxyphenylglucuronide), remaining at this ratio for 0.5 min and then rapidly declining to the start condition. The flow rate was 0.6 ml/min. Peaks of the analytes were detected with a photodiode array detector (PDA, Waters). Analytes, except the glucuronide metabolites, were quantified with a linear calibration curve using peak areas obtained at the compounds’ maximum wavelength. The glucuronide metabolites were, due to the absence of commercially available reference compounds, quantified with the calibration curve and at maximum wavelengths of their parent compound. Differences in the UV absorbance between the parent and the glucuronide conjugate at the selected wavelength were quantified by comparing the peak areas of the parent compound from three different concentrations of the calibration curve with the peak areas of corresponding concentrations of the glucuronide conjugate, which were obtained from a complete glucuronidation of the parent compound in a glucuronidation experiment of 2 h. A noticeable difference between the peak area of the parent compounds and the glucuronide (average of three tested concentrations) was only observed for *p*-fluorophenol (absorbance of conjugate was twofold lower compared to the parent), and this difference was used to correct the measured absorbance of the parent compound from each point of the calibration curve. Fluorescein was quantified with a fluorescence SpectraMax M3 microplate reader (Molecular Devices, USA) with excitation and emission at 495 and 538 nm, respectively.

### Sensitivity analyses of the PBK models

For each PBK model, a local sensitivity analysis was performed to determine influential parameters. To this purpose, each parameter was changed in turn keeping the other ones constant (Chiu et al. 2007). The normalised sensitivity coefficient (SC) was calculated using the algorithm:

$${\text{SC}} = \frac{{\left( {C^{\prime} - C} \right)}}{{\left( {P^{\prime} - P} \right)}}\times\left( {\frac{P}{C}} \right)$$

where *C* is the initial outcome of the model, which in this case is the maximum foetal plasma concentration (*C*_max_). *C*′ is the output of the model after a 1% parameter change. *P* is the initial parameter value, and *P*′ is the parameter value modified by an increase of 1%. The sensitivity analysis was conducted for an oral exposure to a single dose of 2 and 200 mg/kg bw.

### Translation of in vitro effect concentrations in the EST to in vivo effect concentrations

In vitro concentration–response data obtained with the EST were translated to in vivo dose–response values by applying PBK-based reverse modelling, performed as described by Strikwold et al. (2013) with some modifications. To correct for in vivo and in vitro differences in albumin and fat levels, each nominal in vitro effect concentration of *p*-heptyloxyphenol obtained from the EST from Strikwold et al. (2012) was translated to an in vivo effect concentration (EC_*x*_) according to the extrapolation rules of Gülden and Seibert (2003). In the extrapolation rule, a value of 0.99 was used for the fraction of *p*-heptyloxyphenol bound to albumin in the EST, which was obtained from a reported bound fraction of *p*-heptyloxyphenol in the culture medium of the whole embryo culture (WEC) assay (Fisher et al. 1993). In vivo albumin and fat levels that are used in the extrapolation rules were adjusted to values that correspond to embryos/foetuses during mid-/late gestation. An in vivo lipid content of 0.11% was applied based on foetal rat data (GD17) presented by Johansson (1983). An in vivo embryonic/foetal albumin level of 10 mg/ml was used representing albumin levels in mid- and late gestation; GD10 (Yeoh and Morgan 1974) and GD18 (Mcmullin et al. 2008). The vitro lipid fraction and albumin levels resembling the situation in the EST were 0.04% and 4.8 mg/ml, respectively (Verwei et al. 2006). For the other phenols, no correction according the extrapolation rules of Gülden and Seibert (2003) was applied, since it was found that in vitro cytotoxicity of phenol tested with the fibroblast-like embryonic mouse cell line Balb/c 3T3 clone A31 was not affected by differences in bovine serum albumin levels reflecting the in vitro and in vivo situation. It is assumed that this also applies for *p*-methylketophenol and *p*-fluorophenol as these compounds have a comparable log Po:w value as phenol, which is an important factor in albumin binding (Endo and Goss 2011).

Next, the maximum foetal plasma concentrations (*C*_max_) in the PBK model were set equal to the effect concentrations from the EST (which was corrected by the extrapolation rule of Gülden and Seibert (2003) in case of *p*-heptyloxyphenol). In addition, the foetal plasma AUC in the PBK model were set equal to the AUC_0–10d_ from the EST. Applying in vitro PBK-based reverse dosimetry, provided in vivo effective dose levels (ED_*x*_) from which an in vivo dose–response curve, and a BMDL_05_ was derived using the Environmental Protection Agency’s Benchmark Dose Software (BMDS) version 2.6. For each compound, the benchmark dose model selected was the one that provided the best fit determined as described by Strikwold et al. (2012).

### Evaluation of the data

The BMDL_05_ predicted with the in vitro PBK approach were compared to the BMDL_05_ (or NOAEL) derived from an in literature reported in vivo developmental toxicity study with rats that received phenol or *p*-substituted phenol at GD11 Kavlock (1990). The BMDL_05_ values of the in vivo study were derived using the BMDS version 2.6. For each compound, the benchmark dose model was selected that provided the best fit as described by Strikwold et al. (2012). In addition, potency ratios were calculated (potency phenol/potency *p*-substituted phenol) from potency data obtained with the in vitro PBK approach (BMDL_05_), and the in vivo developmental toxicity data (most critical in vivo endpoint) from Kavlock (1990) (BMDL_05_ or NOAEL).

## Results

### In vitro embryotoxicity

**Fig. 2:**
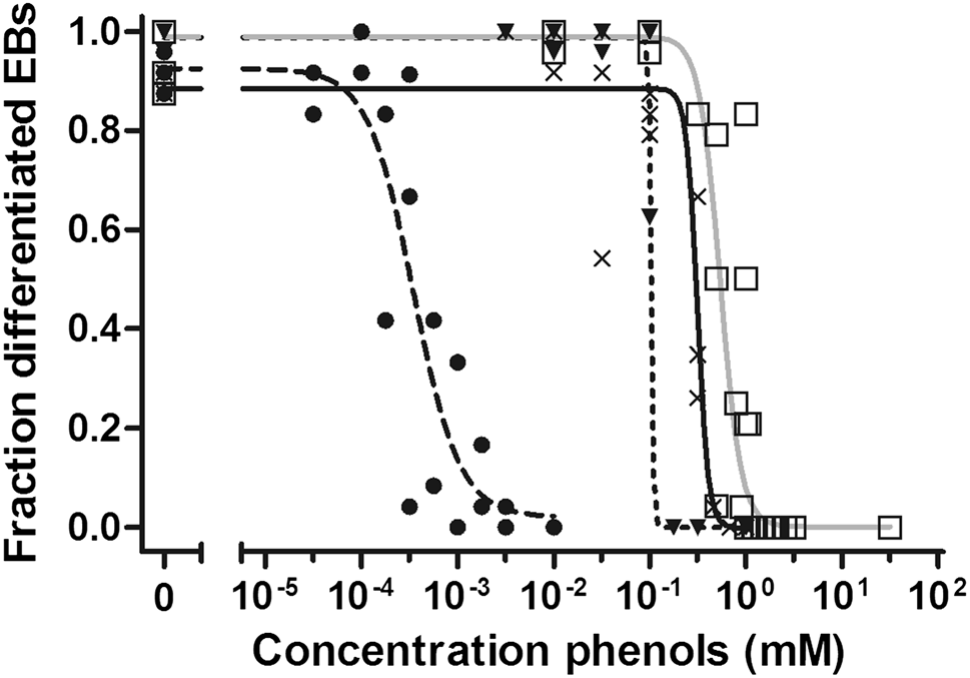
Concentration–response curves for phenol (*grey line*, *open square*), *p*-fluorophenol (*solid line*, *cross symbol*), *p*-heptyloxyphenol (*dashed line*, *filled circle*) and *p*-methylketophenol (*dotted line*, *filled down pointing triangle*) representing the inhibition of differentiation of the embryoid bodies (EBs) by the compound. Data obtained from Strikwold et al. (2012)

Phenol and the *p*-substituted phenols showed a concentration–response-related inhibition of differentiation of ES-D3 cells into beating cardiomyocytes in the EST (Fig. 2) identifying the embryotoxic potential of the different compounds (Strikwold et al. 2012). The highest difference in embryotoxic potential in vitro was observed between phenol and *p*-heptyloxyphenol, with the BMC_50_ value being three orders of magnitude higher for phenol. The differences in BMC_50_ values were much lower between phenol and the other *p*-substituted phenols, including *p*-fluorophenol and *p*-methylketophenol, namely 1.9-fold and 4.9-fold, respectively (Strikwold et al. 2012).

### In silico predictions of biochemical and distribution PBK model parameters

Physicochemical parameters that are used for the prediction of PBK model parameters are outlined in Table 2, and the predicted biochemical and distribution model parameters are presented in Table 3. Notable differences between the compounds are the relatively high log Po:w value of 4.41 for *p*-heptyloxyphenol compared to log Po:w values <1.84 for the other phenols. The tissue–plasma partition coefficients of *p*-heptyloxyphenol are substantially higher than those for the other phenols, especially the fat–plasma partition coefficient which is 84.4 for *p*-heptyloxyphenol and varies between 0.39 and 1.42 for the other phenols. The predicted fraction of phenols unbound to plasma albumin differs from 0.03 for *p*-heptyloxyphenol to 0.38 for phenol.

### In vitro intestinal transport study

Each phenol tested in the Caco-2 transport experiment showed a linear increase of the test concentration in the basal compartment for at least 60 min. The *P*_app_ values of the Caco-2 experiments are presented in Table 4. Transport of each of the test compounds is rapid, and the *P*_app_ values differ at maximum only 1.3-fold between the test compounds, ranging from 51.9 × 10^−6^ cm/s for *p*-methylketophenol to 40.2 × 10^−6^ cm/s for *p*-heptyloxyphenol. The average mass recovery of antipyrine was 93.4%. The mass recovery of phenol, *p*-fluorophenol, *p*-heptyloxyphenol and *p*-methylketophenol was on average 80.2, 78.2, 70.4 and 82.1%, respectively.

**Table 4:** Mean apparent permeability (*P*_app_ ± SD) obtained from Caco-2 and BeWo transport assays, predicted intestinal oral absorption coefficients (ka) and predicted rat transplacental clearance values (CLPL) for the test compounds

Compound	*P*_app_Caco-2_ (10^−6^ cm/s)	ka^a^ (/h)	*P*_app_BeWo_ (10^−6^ cm/s)	CLPL^c^ (l/h)
Phenol	48.4 ± 8.6	7.62^b^	41.6 ± 3.4	0.19
*p*-Fluorophenol	47.7 ± 13.8	7.52	34.2 ± 3.3	0.15
*p*-Heptyloxyphenol	40.2 ± 3.9	6.34	5.9 ± 1.7	0.026
*p*-Methylketophenol	51.9 ± 6.6	8.18	27.5 ± 1.7	0.12
Antipyrine	42.7 ± 2.6	6.73	40.4 ± 3.2	0.18^d^

^a^Predicted oral absorption coefficient (see “Materials and methods” section)

^b^Uptake rate from in situ intestinal perfusion study in rat (Humphrey et al. 1980)

^c^Predicted rat transplacental clearance (see “Materials and methods” section)

^d^Transplacental clearance value obtained from in vivo rat study (Varma and Ramakrishnan 1985). (see “Materials and methods” section)

### In vitro placental transport study

Each phenol tested in the BeWo transport experiment showed a linear increase in concentration in the basal compartment for at least 60 min, after addition of the test compound to the apical side (final concentrations apical compartment 100 and 500 µM). The mass balances showed that >90% of the mass of each compound was conserved in each transport experiment. The *P*_app_ values are presented in Table 4. Transport is rapid for each of the compounds, except for *p*-heptyloxyphenol which showed a *P*_app_ value of 5.9 × 10^−6^ cm/s, which is 6.8-fold lower than the *P*_app_ value of antipyrine. The *P*_app_ values for the other phenols were comparable to antipyrine, showing a 1.03-fold higher and 1.2- and 1.5-fold lower *P*_app_ value compared to the value of the reference compound antipyrine for phenol, *p*-fluorophenol and *p*-methylketophenol, respectively. The transport of the paracellular control fluorescein was on average 11-fold lower compared to the passive transcellular control antipyrine indicating the integrity of the monolayer.

An interesting observation was that the *P*_app_ value for *p*-heptyloxyphenol obtained from the BeWo experiment was 6.8-fold lower than the *P*_app_ value from the Caco-2 experiment, while the *P*_app_ values for the other phenols were less than twofold lower in the BeWo assay, and the *P*_app_ value from the BeWo experiment for antipyrine was only 1.1-fold lower than the *P*_app_ from the Caco-2 experiment.

### In vitro glucuronidation of *p*-substituted phenols by rat liver microsomes

**Fig. 3:**
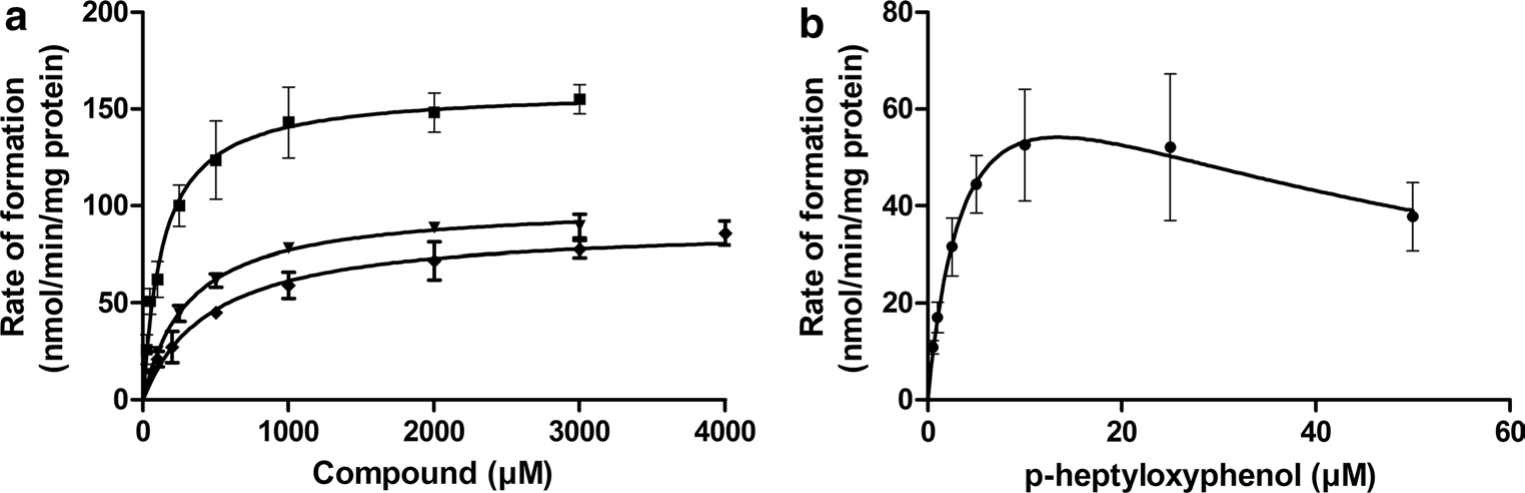
Concentration-dependent formation of the glucuronide conjugate of phenol (*filled diamond*), *p*-fluorophenol (*filled down pointing triangle*), *p*-methylketophenol (*filled square*) (**a**) and *p*-heptyloxyphenol (*filled circle*) (**b**) in incubations with rat liver microsomes. *Individual symbols* represent mean activities of 3–4 independently performed experiments ±SD

Results from the incubation experiments showed that rat liver microsomes were able to metabolise the *p*-substituted phenols to their glucuronide conjugates. Metabolism of the *p*-substituted phenols followed Michaelis–Menten kinetics (Fig. 3). The apparent *K*_m_ and *V*_max_ values of the rat liver microsomes and the (scaled) catalytic efficiencies are presented in Table 5. The compound *p*-heptyloxyphenol was far more efficiently converted by liver microsomes than the other phenols. The scaled catalytic efficiency (l/h/g liver) for glucuronidation of *p*-heptyloxyphenol was 145-fold higher than that of phenol. The scaled catalytic efficiencies of *p*-fluorophenol and *p*-methylketophenol were respectively 1.7-fold and sixfold higher than that of phenol.

**Table 5:** Kinetic constants *K*_m_ and *V*_max_ ± SD, and catalytic efficiencies of the formation of the glucuronide metabolite of the phenols in rat liver microsomes

Compound	${K_{\text {m(app)}}}^\text a$	${V_{\text {max(app)}}}^\text b$	Catalytic efficiency^c^	Scaled ${V_{\text {max(app)}}}^\text d$	Scaled catalytic efficiency^e^
Phenol	465 ± 66	90 ± 4	0.19	205	0.44
*p*-Fluorophenol	314 ± 16	101 ± 1.6	0.32	231	0.74
*p*-Heptyloxyphenol	2.1 ± 0.6	60 ± 4.5	28.0	137	63.8
*p*-Methylketophenol	138 ± 17	160 ± 4.4	1.16	365	2.65

^a^µM

^b^nmol/min/mg microsomal protein

^c^ *V*_max_/*K*_m_ (ml/min/mg protein in rat liver microsomes)

^d^µmol/h/g liver

^e^l/h/g liver

### Sensitivity analysis

**Fig. 4:**
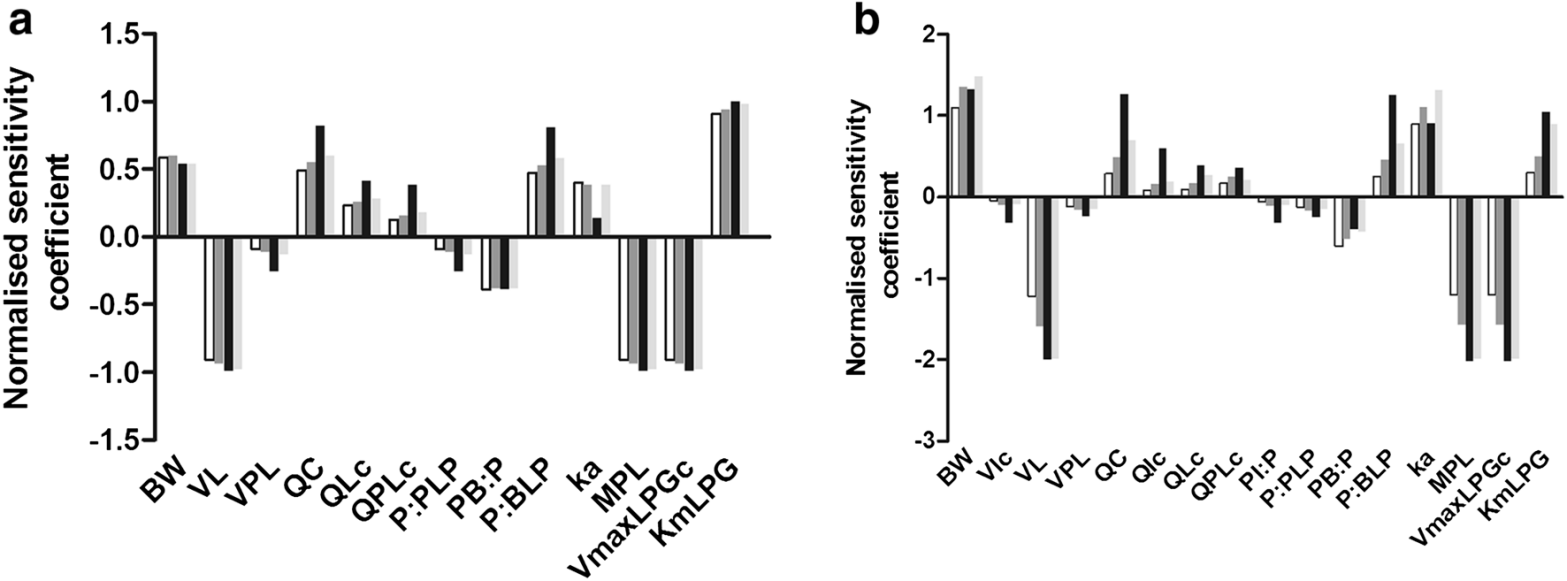
Normalised sensitivity coefficients for parameters of the PBK model for phenol (*white bars*), *p*-fluorophenol (*dark grey bars*), *p*-heptyloxyphenol (*black bars*) and *p*-methylketophenol (*light grey bars*) based on foetal *C*_max_ values from a single oral dose of 2.0 mg/kg bw (**a**) and 200 mg/kg bw (**b**). Normalised sensitivity coefficients ≥0.2 are presented. *BW* body weight, *VIc* fraction intestinal tissue, *VL* fraction liver tissue, *VPL* volume placental tissue, *QC* cardiac output, *QIc* fraction intestinal flow, *QLc* fractional liver flow, *QPLc* fractional placental flow, *PI:P* partition coefficient intestine:plasma, *PPL:P* partition coefficient placenta:plasma, *PB:P* partition coefficient body remaining:plasma, *PBL:P* partition coefficient blood:plasma, *ka* oral absorption coefficient, *MPL* liver microsomal protein yield, *VmaxLPGc* unscaled maximum rate of glucuronidation of phenols in liver, *KmLPG* Michaelis–Menten constant for glucuronidation of phenols in liver

The results of the sensitivity analysis performed at a high oral dose of 200 mg/kg bw (Fig. 4) indicated that the most influential parameters for the phenol models are the body weight, cardiac output, volume of the liver, parameters related to glucuronidation in the liver (*V*_max_, *K*_m_ and liver microsomal protein yield), the oral absorption coefficient and the partition blood-plasma coefficient. The parameter *K*_m_ was of higher influence in the models of *p*-methylketophenol and *p*-heptyloxyphenol than in the other phenol models. In the model for *p*-heptyloxyphenol, the cardiac output and parameters related to the intestine (volume intestine, flow to intestine and partition coefficient intestinal tissue–plasma) are more influential than in the models of the other phenols. Parameters related to the foetal/placental compartment do not highly influence the model outcome.

In general, the model outcomes are sensitive to similar parameters at a low oral dose of 2 mg/kg bw when compared to the analysis at 200 mg/kg bw. Parameters related to the intestine in the model for *p*-heptyloxyphenol are not of influence at a low dose of 2 mg/kg bw. Moreover, the values of sensitivity coefficients of the phenols for ka, body weight and volume of the liver, derived at a low dose of 2 mg/kg bw, are lower than the sensitivity coefficients derived at 200 mg/kg bw. The sensitivity coefficients for the microsomal protein content and the *V*_max_ for glucuronidation in the liver for *p*-fluorophenol, *p*-heptyloxyphenol and *p*-methylketophenol are lower than the sensitivity coefficients for these parameters derived at 200 mg/kg bw, while the sensitivity coefficients for phenol and *p*-fluorophenol for *K*_m_ are higher when compared to the sensitivity coefficients derived at 200 mg/kg bw.

### Translation of in vitro effect concentrations in the EST to in vivo dose levels

**Fig. 5:**
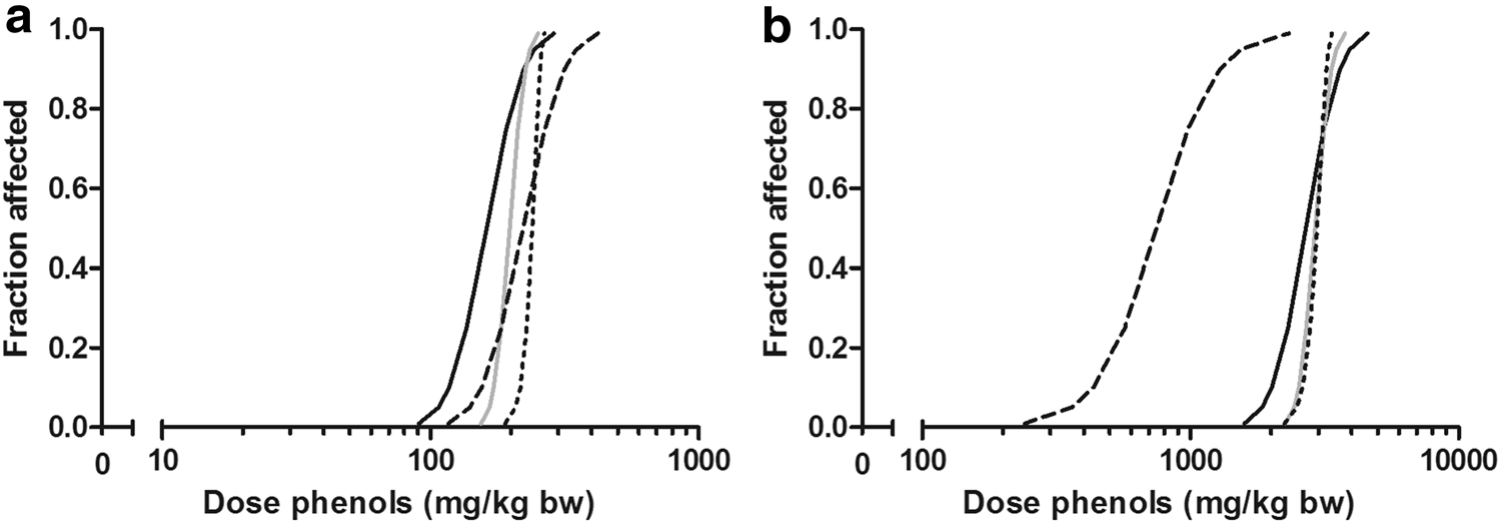
In vivo dose–response curves for developmental toxicity of phenol (*solid black line*), *p*-fluorophenol (*solid grey line*), *p*-heptyloxyphenol (*dashed line*) and *p*-methylketophenol (*dotted line*) in rat predicted by in vitro PBK-based reverse dosimetry. **a** Represents the predicted dose–response curves based on the nominal test concentration of the phenols relating *C*_max_ to developmental toxicity. **b** Represents the predicted dose–response curves relating the AUC_0–10d_ of the phenols to developmental toxicity

In a first step the in vitro concentrations of *p*-heptyloxyphenol were converted to equivalent plasma concentrations using extrapolation rules of Gülden and Seibert (2003). Only a very small difference was observed between the in vitro effect concentrations from the EST and the estimated equivalent plasma concentrations, with the latter being 2.1-fold higher than the in vitro effect concentrations. In a second step, the in vivo effect concentrations were translated to external in vivo oral dose values using the PBK models thus defining a dose–response curve from which a PoD could be derived. The predicted dose–response curves based on *C*_max_ or the AUC_0–10d_ are presented in Fig. 5. The BMDL_05_ values for the phenols predicted by the in vitro PBK approach using *C*_max_ and the AUC_0–10d_ from the EST as a dose metric for reverse dosimetry are outlined in Table 6, together with the BMDL_05_ values that were predicted from in vivo developmental toxicity data reported in the literature. The in vivo experimental data and the PBK model-based predictions were representative for exposure at GD11 since in the in vivo experimental study exposure was at GD11 and for the predictions the PBK model was defined using physiological parameters representative for GD11. Comparing the BMDL_05_ values that were predicted with the in vitro PBK approach with the BMDL_05_ values derived from in vivo data indicates that the BMDL_05_ that is based on *C*_max_ as a dose metric represents the in vivo BMDL_05_ better than the BMDL_05_ based on the AUC_0–10d_ for phenol and *p*-fluorophenol, while for *p*-methylketophenol both the BMDL_05_ based on *C*_max_ and AUC_0–10d_ seem to represent the in vivo BMDL_05_ well. The difference between the BMDL_05_ values predicted with the in vitro PBK approach based on *C*_max_ of the EST as a dose metric and the BMDL_05_ values obtained with in vivo developmental toxicity data vary less than 3.6-fold for these three phenols. For *p*-heptyloxyphenol, however, the BMDL_05_ value based on AUC_0–10d_ of the EST as a dose metric represents the in vivo BMDL_05_ somewhat better (1.7-fold difference) than the predicted BMDL_05_ based on *C*_max_ (4.2-fold difference).

**Table 6:** BMDL_05_ values (mg/kg bw) for developmental toxicity of the phenols predicted with the in vitro PBK approach and BMDL_05_ values derived from in vivo developmental toxicity data reported in the literature

Compound	In vitro PBK approach		In vivo
	BMDL_05_*C*max_	BMDL_05_AUC_	BMDL_05_ (or NOAEL)^a^
Phenol	92	1603	333
*p*-Fluoro	135	1939	183
*p*-Heptyloxy	115	281	484
*p*-Methylketo	194	2265	632

^a^Derived from in vivo developmental toxicity data (most critical endpoint) from Kavlock (1990)

**Fig. 6:**
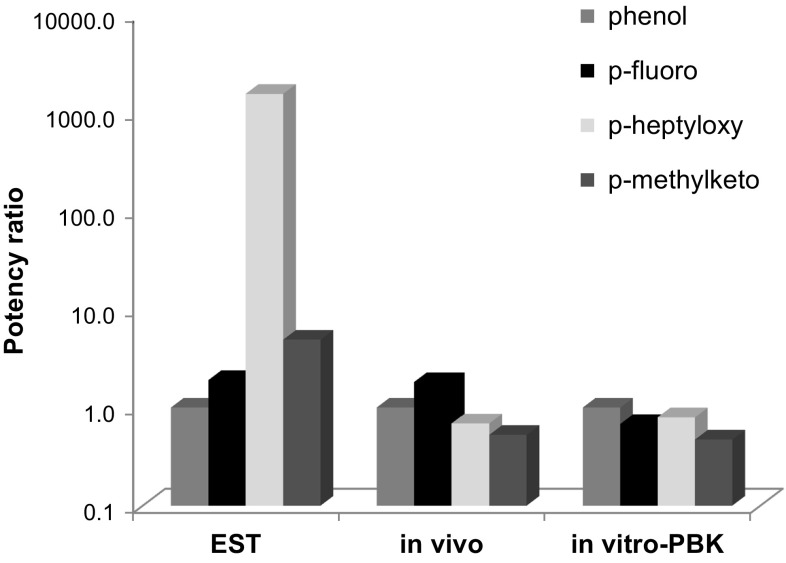
Potency of *p*-substituted phenols relative to phenol expressed as a potency ratio (potency ratio = potency phenol/potency *p*-substituted phenol), specified for the in vitro PBK approach (BMDL_05_) using *C*_max_ as dose metric, the in vivo data (most critical endpoint) from Kavlock (1990) (BMDL_05_ or NOAEL) and the EST (BMC_50_)

The potency ratios between phenol and *p*-fluorophenol, *p*-heptyloxyphenol and *p*-methylketophenol calculated based on the BMDL_05_ values obtained with the in vitro PBK approach are graphically presented in Fig. 6, together with the potency ratios obtained from the in vivo developmental toxicity study of Kavlock (1990) and the EST. The difference between the most and the least potent test compound in the EST, *p*-heptyloxyphenol and phenol was three orders of magnitude (Strikwold et al. 2012), which does not reflect the potency ratio obtained from the in vivo developmental toxicity study which shows a small potency difference between the phenols (maximum 0.5-fold for the potency ratio based on the in vivo BMDL_05_ or NOAEL). From Fig. 6, it can be seen that the large difference in the toxic potency that was observed in the EST for *p*-heptyloxyphenol compared to phenol was greatly diminished, namely from 1553-fold in the EST to 0.8-fold in the in vitro PBK approach when based on the *C*_max_. The potency ratio between phenol and *p*-fluorophenol and *p*-methylketophenol was changed from 1.9- to 0.7-fold and from 4.9- to 0.5-fold, respectively.

## Discussion and conclusion

Translation of in vitro toxicity data to in vivo toxicity values is highly relevant in order to use in vitro data in the regulatory risk assessment of chemicals. In previous studies it was demonstrated that PBK-based reverse dosimetry converting in vitro concentration–response values to in vivo dose–response data could successfully be applied to predict a PoD for phenol (Strikwold et al. 2013), for all-trans-retinoic-acid (Louisse et al. 2015) and for some glycol-ethers (Louisse et al. 2010). The aim of the present study was to investigate whether PBK-based reverse dosimetry could be used to translate in vitro embryotoxicity data obtained with the EST for a series of phenols (Strikwold et al. 2012) to in vivo developmental toxic potency values for the rat, using only in silico- and in vitro-derived (kinetic) parameters and data from the literature, and whether this approach could overcome differences in in vitro and in vivo relative potencies of different phenolic congeners observed by Strikwold et al. (2012). The PoDs predicted with the in vitro PBK approach differed only 3.6-fold from the PoDs derived from in vivo data from literature, when *C*_max_ of the EST was considered to be the most appropriate dose metric for in vitro PBK-based reverse dosimetry for phenol and *p*-fluorophenol and the AUC_0–10d_ for *p*-heptyloxyphenol, while both *C*_max_ and AUC_0–10d_ were appropriate metrics for *p*-methylketophenol. The large difference between the in vitro-derived relative potency and the in vivo-derived relative potency of *p*-heptyloxyphenol was reduced from three orders of magnitude for the EST data as such (Strikwold et al. 2012) to <2.1-fold after applying PBK-based reverse dosimetry to these EST data.

Results from our in silico- and in vitro-derived (kinetic) parameters together with PBK modelling provide insight into the possible factors underlying the relative low toxic potency for *p*-heptyloxyphenol in vivo (Kavlock 1990) compared to the relatively high observed embryotoxic potency in vitro in the EST (Strikwold et al. 2012). This discrepancy may be due to three major factors that play a role in vivo but not in the EST in vitro model, including (1) the relatively rapid metabolism of *p*-heptyloxyphenol by glucuronidation, (2) the relatively low placental transport of *p*-heptyloxyphenol compared to the other phenols and (3) the relatively high tissue-plasma partition coefficients of *p*-heptyloxyphenol. Of these three aspects, the rapid glucuronidation of the toxic parent compound *p*-heptyloxyphenol has the largest contribution to the improved relative potency prediction for this compound. The contribution of metabolism in diminishing the toxicity of *p*-heptyloxyphenol was also observed in the WEC assay where the embryotoxic potency of *p*-heptyloxyphenol was greatly reduced when hepatocytes were added to the WEC assay (Oglesby et al. 1992).

The affinity constant *K*_m_ for glucuronidation of the phenols in the liver was found to be an influential kinetic parameter in the sensitivity analysis. The *K*_m_ of *p*-heptyloxyphenol was much lower than the *K*_m_ of the other tested phenols, resulting in a very rapid glucuronidation and elimination of the toxic parent compound. The high lipophilicity of *p*-heptyloxyphenol may explain the high affinity for the enzyme.

Transport across the placenta was determined with the BeWo cell line, and provided high *P*_app_ values for each of the test compound, except for *p*-heptyloxyphenol for which the *P*_app_ value was 7.1-fold lower compared with phenol. The high *P*_app_ values are in line with in vivo studies in rats reporting that simple phenolic compounds may readily pass the placenta (Abu-Qare et al. 2000; Gray and Kavlock 1990). In general, cellular permeability increases with increasing lipophilicity, until a certain threshold (Waterhouse 2003; Wils et al. 1994). It has been demonstrated in vitro with intestinal cells that transport may decrease for compounds with an octanol–buffer distribution coefficient >3000 (Wils et al. 1994), which corresponds to our observations for *p*-heptyloxyphenol [logP:ow = 25,704 (ACD/Labs 2015)] in the Caco-2 and the BeWo assays. Interestingly, the *P*_app_ value of *p*-heptyloxyphenol in the BeWo transport experiment was 7.1-fold lower than the *P*_app_ value of phenol while this was only 1.2-fold in the Caco-2 assay. The observed difference may be due to binding of *p*-heptyloxyphenol to albumin that is present in the apical medium of the BeWo assay but not in the apical medium of Caco-2 assay. These albumin levels were selected to reflect physiological conditions. The predicted *P*_app_ value in the BeWo assay for *p*-heptyloxyphenol may be somewhat lower than the in vivo value, as Li et al. (2013) observed that the relative *P*_app_ value of the highly albumin bound compound ketoprofen was 3.4-fold lower in the BeWo system with albumin, compared to the relative *P*_app_ determined with the ex vivo perfusion system that also included albumin. This difference possibly originates from differences in the fluid dynamics of both systems, which was static in the BeWo assay and is dynamic in the ex vivo perfusion system (Li et al. 2013).

Incorporating permeability data obtained from the BeWo transport system to semi-quantitatively predict placental transfer in the PBK models in the present study presents an approach that has not been applied in PBK modelling before. The BeWo transport system was evaluated to be a valuable in vitro model to predict transport of compounds across the placenta (Li et al. 2013). Nonetheless, this approach may be explored further, investigating the applicability domain of the BeWo assay with respect to different chemical classes, i.e. lipophilic compounds, as well as the time of pregnancy to augment its utility.

The present study predicted the embryotoxic potency of the phenols with physiological parameters in the PBK model selected for GD11 because for this time point in vivo developmental toxicity data for evaluation of the predictions were available. The EST was used to represent a sensitive in vitro endpoint for developmental toxicity (Genschow et al. 2004), which ideally may represent the sensitivity of the embryo at the critical window for toxicity of the *p*-substituted phenols. Parameterisation of the PBK model for other gestational days is possible. Calculations with parameters adjusted to GD20 (data not shown) indicate little differences with GD11, providing a 1.2- to 2.3-fold higher BMDL_05_ value for predictions made with the PBK parameters for GD20 than for GD11 for the different phenols. Based on these results, the predictions for GD11 may be regarded to represent a sensitive period.

In our study we extrapolated each nominal effective concentration tested in the EST to an in vivo effective dose value using PBK-based reverse dosimetry, from which an in vivo dose–response curve and BMDL_05_ values were derived with the BMDS software. Sometimes only a single BMC_*x*_ is available (often a BMC_50_) and extrapolating this single BMC_50_ to a BMD_50_ with reverse dosimetry will likely result in almost similar BMD_50_ values as would be obtained with our approach when kinetics are linear, but may deviate to a larger extent in case of nonlinear kinetics. Moreover, extrapolating just the BMC_50_ value to a BMD_50_ value may bring other difficulties as we cannot extrapolate this value easily to a PoD that would be relevant for the risk assessment, like a BMDL_05_ or BMDL_10_. Therefore in our study, we extrapolated each nominal effective concentration to an in vivo effective dose value as described above.

In conclusion, PBK models were developed for a series of phenols, using in vitro, in silico data and data obtained from the literature only. Applying the in vitro PBK-based reverse dosimetry approach to overcome kinetic differences between the in vitro toxicity test system and the in vivo situation resulted in an improved prediction of the in vivo developmental toxic potency for this series of phenols. This approach has even overcome the large disparities that were observed between the in vitro and the in vivo relative potencies of *p*-heptyloxyphenol. Herewith, we provide another proof of principle that integrating in vitro toxicity data and PBK-based reverse dosimetry may be a promising approach for prospective toxicological safety evaluations of compounds, without performing animal testing.

## Electronic supplementary material

Below is the link to the electronic supplementary material.

Supplementary material 1 (PDF 117 kb)
