# Supplementary material for: Integrating in vitro data and physiologically based kinetic (PBK) modelling to assess the in vivo potential developmental toxicity of a series of phenols
Source: Arch Toxicol. 2016 Nov 4;91(5):2119–33. doi: 10.1007/s00204-016-1881-x (PMC5399052; doi:10.1007/s00204-016-1881-x)
Supplement: Supplementary file 1 — Supplementary material 1 (PDF 117 kb) [file 204_2016_1881_MOESM1_ESM.pdf]

Supplementary data A

Mass balance algorithms and parameter specifications of the PBK model for phenol and p-substituted phenols in the rat

The algorithms are similar for phenol, p-fluorophenol, p-heptyloxyphenol and p-methylketophenol. An example for the compound phenol is presented.

**Compound**	**abbreviation**
phenol	ph
phenylglucuronide	pg

**Compartment (Tissue (T))**	**abbreviation**
Small intestine	I
Liver	L
Fat	F
Placenta	PL
Foetus	FE
Remaining body	B
Arterial	A
Venous	V
Blood	BL

**Variable**	**Unit**	**abbreviation**
Blood flow rate to tissue	$l\ h^{-1}$	Q(T)
Cardiac output	$l\ h^{-1}$	QC
Concentration phenol in tissue or blood	µM	$C(T)_{ph}$
Partition coefficient tissue:plasma phenol	-	$P(T)_{ph}$
Volume of tissue or blood	l	V(T)

Amount phenol in tissue or blood	μmol	$A(T)_{ph}$
Maximum rate of formation metabolite (m) in tissue	μmol h^{-1}	$Vmax(T)_{m}$
Michaelis-Menten constant for formation metabolite (m) in tissue	μM	$Km(T)_{m}$
Uptake rate phenol intestine	h^{-1}	ka
Amount phenol taken up from the gut lumen	μmol	$Uptake_{ph}$
Amount phenol remaining in the gut lumen	μmol	AGL_{ph}
Clearance placenta	l h^{-1}	CLPL

Small intestine

$$\frac{dAI_{ph}}{dt} = \frac{dUptake_{ph}}{dt} + QI * \left(CA_{ph} - \frac{CI_{ph}}{PI_{ph}} * PBL_{ph}\right)$$

Uptake phenol from gut lumen

$$\frac{dUptake_{ph}}{dt} = -\frac{dAGL_{ph}}{dt} = ka * AGL_{ph}$$

$$AGL_{ph}\,(0) = Oral\ dose$$

$$CI_{ph} = \frac{AI_{ph}}{VI}$$

Liver compartment

$$\frac{dAL_{ph}}{dt} = QL * CA + \ QI * \frac{CI_{ph}}{PI_{ph}} * PBL_{ph} - \ (QL + QI) * \frac{CL_{ph}}{PL_{ph}} * PBL_{ph} - \frac{VmaxL_{pg} * \frac{CL_{ph}}{PL_{ph}}}{KmL_{pg} + \frac{CL_{ph}}{PL_{ph}}}$$

$$CL_{ph} = \frac{AL_{ph}}{VL}$$

Fat compartment

$$\frac{dAF_{ph}}{dt} = QF * \left(CA_{ph} - \frac{CF_{ph}}{PF_{ph}} * PBL_{ph}\right)$$

$$CF_{ph} = \frac{AF_{ph}}{VF}$$

Placental/foetal compartment

Phenol in placental compartment

$$\frac{dAPL_{ph}}{dt} = QPL * \left(CA_{ph} - \frac{CPL_{ph}}{PPL_{ph}} * PBL_{ph}\right) + CLPL * \left(\frac{CFE}{PFE} - \frac{CPL}{PPL}\right)$$

$$CPL_{ph} = \frac{APL_{ph}}{VPL}$$

Phenol in foetal compartment

$$\frac{dAFE_{ph}}{dt} = CLPL * \left(\frac{CPL}{PPL} - \frac{CFE}{PFE}\right)$$

$$CFE_{ph} = \frac{AFE_{ph}}{VFE}$$

Remaining body tissue

$$\frac{dAB_{ph}}{dt} = QB * \left(CA_{ph} - \frac{CB_{ph}}{PB_{ph}} * PBL_{ph}\right)$$

$$CB_{ph} = \frac{AB_{ph}}{VB}$$

Arterial blood compartment

$$CA_{ph} = CV_{ph}$$

Venous blood compartment

$$\frac{dAV_{ph}}{dt} = (QL + QI) * \frac{CL_{ph}}{PL_{ph}} * PBL_{ph} + QF * \frac{CF_{ph}}{PF_{ph}} * PBL_{ph} + QPL * \frac{CPL_{ph}}{PPL_{ph}} * PBL_{ph} + QB * \frac{CB_{ph}}{PB_{ph}} * PBL_{ph}$$

$$-QC * CV_{ph}$$

$$CV_{ph} = \frac{AV_{ph}}{VV}$$

Note: the maternal circulation is via blood, but exchange between mother and foetus is modelled to occur via plasma because that represents the BeWo system better. For the reverse dosimetry, the foetal plasma was set equal to the concentration in the EST.
